# Virtual Reality-Based Early Neurocognitive Stimulation in Critically Ill Patients: A Pilot Randomized Clinical Trial

**DOI:** 10.3390/jpm11121260

**Published:** 2021-11-29

**Authors:** Guillem Navarra-Ventura, Gemma Gomà, Candelaria de Haro, Mercè Jodar, Leonardo Sarlabous, David Hernando, Raquel Bailón, Ana Ochagavía, Lluís Blanch, Josefina López-Aguilar, Sol Fernández-Gonzalo

**Affiliations:** 1 Critical Care Center, Hospital Universitari Parc Taulí, Institut d’Investigació i Innovació Parc Taulí I3PT, Universitat Autònoma de Barcelona, 08208 Sabadell, Spain; ggoma@tauli.cat (G.G.); cdeharo@tauli.cat (C.d.H.); lsarlabous@tauli.cat (L.S.); aochagavia@tauli.cat (A.O.); lblanch@tauli.cat (L.B.); jlopeza@tauli.cat (J.L.-A.); msfernandez@tauli.cat (S.F.-G.); 2 Department of Mental Health, Hospital Universitari Parc Taulí, Institut d’Investigació i Innovació Parc Taulí I3PT, Universitat Autònoma de Barcelona, 08208 Sabadell, Spain; 3 Centro de Investigación Biomédica en Red de Enfermedades Respiratorias (CIBERES), Instituto de Salud Carlos III, 28029 Madrid, Spain; 4 Department of Neurology, Hospital Universitari Parc Taulí, Institut d’Investigació i Innovació Parc Taulí I3PT, Universitat Autònoma de Barcelona, 08208 Sabadell, Spain; mjodar@tauli.cat; 5 Centro de Investigación Biomédica en Red de Salud Mental (CIBERSAM), Instituto de Salud Carlos III, 28029 Madrid, Spain; 6 Department of Clinical and Health Psychology, Universitat Autònoma de Barcelona, 08193 Bellaterra, Spain; 7 Instituto Universitario de Investigación en Ingeniería de Aragón, Universidad de Zaragoza, 50018 Zaragoza, Spain; dhernand@unizar.es (D.H.); rbailon@unizar.es (R.B.); 8 Instituto de Investigación Sanitaria Aragón, Universidad de Zaragoza, 50009 Zaragoza, Spain; 9 Centro de Investigación Biomédica en Red de Bioingeniería, Biomateriales y Nanomedicina (CIBERBBN), Instituto de Salud Carlos III, 28029 Madrid, Spain

**Keywords:** critical illness, digital therapy, prevention, post-intensive care syndrome, working memory

## Abstract

This study focuses on the application of a non-immersive virtual reality (VR)-based neurocognitive intervention in critically ill patients. Our aim was to assess the feasibility of direct outcome measures to detect the impact of this digital therapy on patients’ cognitive and emotional outcomes. Seventy-two mechanically ventilated adult patients were randomly assigned to the “treatment as usual” (TAU, *n* = 38) or the “early neurocognitive stimulation” (ENRIC, *n* = 34) groups. All patients received standard intensive care unit (ICU) care. Patients in the ENRIC group also received adjuvant neurocognitive stimulation during the ICU stay. Outcome measures were a full neuropsychological battery and two mental health questionnaires. A total of 42 patients (21 ENRIC) completed assessment one month after ICU discharge, and 24 (10 ENRIC) one year later. At one-month follow-up, ENRIC patients had better working memory scores (*p* = 0.009, *d* = 0.363) and showed up to 50% less non-specific anxiety (11.8% vs. 21.1%) and depression (5.9% vs. 10.5%) than TAU patients. A general linear model of repeated measures reported a main effect of group, but not of time or group–time interaction, on working memory, with ENRIC patients outperforming TAU patients (*p* = 0.008, η_p_^2^ = 0.282). Our results suggest that non-immersive VR-based neurocognitive stimulation may help improve short-term working memory outcomes in survivors of critical illness. Moreover, this advantage could be maintained in the long term. An efficacy trial in a larger sample of participants is feasible and must be conducted.

## 1. Introduction

Approximately half of intensive care unit (ICU) survivors develop post-intensive care syndrome (PICS) [1], which comprises a set of acquired physical, cognitive, and emotional deficits that negatively affect the quality of life of patients and their families [2]. These disorders can drastically alter the ability to perform activities of daily living normally [3] and even prevent patients from returning to work [4]. In addition, they are associated with high medical and financial costs [5]. However, there is evidence that their severity can be mitigated by preventive interventions during the early stages of critical illness [1].

The cognitive impairment associated with PICS resembles that of mild-to-moderate dementia [6] and may persist for years after discharge [7]. This impairment can adopt different phenotypes depending on the severity of dysfunction and the cognitive domains affected, being especially pronounced in memory, executive functions, and processing speed [8]. Several variables, such as older age, female gender, mechanical ventilation (MV), delirium, and deep sedation have been associated with an increased risk of cognitive sequelae. Conversely, greater cognitive reserve, i.e., the brain’s resistance to pathological changes [9], may be a protective factor [10,11,12].

ICUs are places for the care of critically ill, unstable, and recoverable patients who are at risk of dying. Therefore, they are a potentially hostile environment for a vulnerable patient. In addition to the physical stress associated with critical illness and interventions (e.g., MV), there are physiological (e.g., delirium) and psychosocial factors (e.g., noise, ambient light, restricted mobility, and social isolation) that can adversely affect patient comfort and well-being. Consequently, patients can also experience a strong emotional impact related to the ICU environment, their critical illness, and associated sequelae, and suffer non-specific anxiety, post-traumatic stress disorder (PTSD), and depression [13]. Stressors such as longer ICU and hospital stay, prolonged MV, negative or delusional experiences during ICU admission, and functional disability after discharge are risk factors for the development of these disorders [5,14].

Interest in post-ICU sequelae and how to prevent them has increased markedly in recent years [15,16]. The ABCDEF bundle is an evidence-based clinical guideline that provides recommendations to improve patient recovery and outcomes during the ICU stay [17]. Common measures to mitigate PICS include optimized delirium management [18]; early mobilization [19]; and, in some cases, occupational therapy [20]. Over the years, these measures have expanded to include in situ neurocognitive stimulation [21] and stress and anxiety reduction [22]. However, ICU patients are usually bedridden and often unable to communicate verbally, especially when undergoing MV. In these cases, classical neurocognitive and psychotherapeutic interventions are particularly difficult to apply. To address this limitation, several technology-based solutions have been proposed [23].

Virtual reality (VR) refers to immersive, multisensory, viewer-centered, 3D computer-generated, interactive environments created by combining different technologies. Cognitive and emotional therapies based on VR techniques have achieved promising results in patients with neurocognitive and mental health disorders [24,25], but reports on their use in critically ill patients are scarce [26,27,28,29]. Thus far, most studies in critically ill patients have used immersive systems that provide a full stereoscopic experience through the use of various sensory output devices, such as head-mounted displays [26,28,29]. Other studies have used non-immersive systems that employ computer displays or large projection screens that are less expensive but do not provide a full stereoscopic experience [27]. Despite these differences, all have used simulated environments inspired by the real world and based on images and sounds from nature, as these are considered the most appropriate scenarios to help patients abstract from the ICU environment and their critical illness. However, none have been designed specifically for MV patients, who are at high risk of post-ICU sequelae due to the cross-talks between lung and brain [2,11,13,14].

Patient-centered care aims to make patients feel known, respected, and well-informed, actively involved in their own care and in decisions related to their illness and recovery process. With this in mind, we developed the Early Neurocognitive Rehabilitation in Intensive Care (ENRIC) platform, an innovative non-immersive VR-based early neurocognitive intervention designed specifically for the ICU environment and the MV patient. In a first proof-of-concept (PoC) study, we already demonstrated the feasibility of this intervention in terms of safety, tolerability, and potential efficacy in stimulating the brain using a surrogate outcome measure, such as heart rate variability [30]. However, its potential efficacy in mitigating post-ICU sequelae using direct outcome measures, such as neuropsychological testing and mental health questionnaires, has yet to be evaluated. A more precise understanding of how and to what extent ENRIC therapy helps improve cognitive and emotional outcomes in survivors of critical illness may contribute to a better approach to disease management that also addresses comfort and well-being.

Following CONSORT guidelines for non-pharmacological [31] and pilot trials [32], the overall purpose of this second PoC study was to assess the feasibility of direct outcome measures to detect differences between stimulated vs. non-stimulated groups and to provide data (e.g., mean, standard deviation, 95% confidence interval) for the design of future efficacy trials in larger samples of participants. To this end, we set two specific objectives: first, to evaluate the impact of ENRIC therapy on patients’ cognitive and emotional outcomes one month after ICU discharge, and second, to analyze whether or not the effect of this intervention is maintained one year later.

## 2. Materials and Methods

### 2.1. Study Design

This is a prospective, randomized, non-blind, two-arm, pilot clinical trial that was conducted between November 2015 and September 2020. The study was approved by the Ethics Committee of the Parc Taulí University Hospital (Catalonia, Spain; #2013/067), registered in ClinicalTrials.gov (NCT02078206) and carried out in accordance with the latest version of the Declaration of Helsinki.

### 2.2. Sample Size

**Figure 1:**
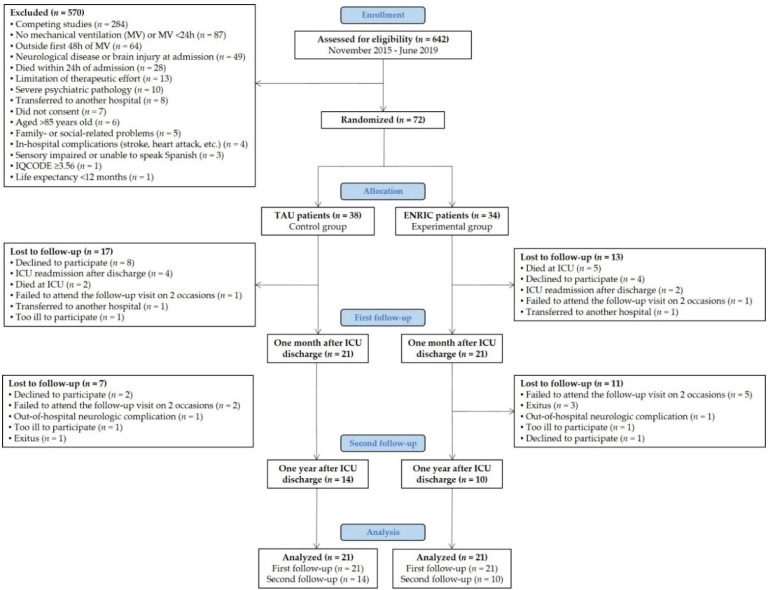
Flow diagram. TAU—treatment as usual, ENRIC—early neurocognitive stimulation, ICU—intensive care unit, IQCODE—informant questionnaire on cognitive decline in the elderly. Over a four-year period, 642 ICU patients were screened for eligibility, but 570 met at least one exclusion criterion. Of the 72 eligible patients, 38 (52.8%) were randomly assigned to the TAU group and 34 (47.2%) to the ENRIC group. Forty-two patients (58.3%) completed the cognitive and emotional evaluation one month after ICU discharge. Twenty-four patients (57.1%) were re-evaluated one year later.

The minimum sample size for a pilot randomized clinical trial (RCT) should be 9% of the number required in the main study [33]. The sample size of a full RCT calculated with the G*Power software v3.1.9.4, assuming a conservative effect size of 0.20 at a study power of 0.80 and with an alpha error of 0.05 would be 778 subjects (786 according to [33]). Therefore, 72 critically ill patients were recruited for this pilot RCT (Figure 1).

### 2.3. ENRIC Technology Features

The ENRIC platform, designed by a multidisciplinary team of neuropsychologists, critical care physicians, nurses, and other biomedical researchers [30], features stimulation software with various cognitive exercises, including passive, guided observation, selective attention, and working memory exercises, specifically designed or adapted to the ICU environment and MV patient. All cognitive exercises are based on previous cognitive rehabilitation programs that have proven effective in improving cognitive outcomes in patients with neurocognitive and mental health disorders [34,35]. Using a central processing unit, a flat screen TV, and a Kinect^®^ motion sensor to detect the movement of the patient’s arms and hands, the stimulation software places patients in a relaxing environment (tropical island) composed of 4 scenarios with real nature sounds (wheat field, beach, forest, and mountain landscape) in which they can walk accompanied by a virtual avatar. All scenarios are similar to previous environments that have proven effective in reducing stress and anxiety in critically ill patients [26,27,28,29]. The route through the virtual world and the order of the cognitive exercises is initially predefined; however, the therapist can modify the route, reconfigure the order of appearance of the exercises, or jump from one exercise to another using commands. Together with the therapist, the virtual avatar orientates the patients in time and delivers instructions, while encouraging them to do the cognitive exercises and to relax (see Supplementary Files S1 and S2).

### 2.4. Participants and Procedure

Patients were recruited from the ICU of the Parc Taulí University Hospital, a mixed medical/surgical ICU with 16 beds in single rooms. All subjects were aged 18–85 years old and had undergone ≥24 h of invasive MV. Exclusion criteria were prior cognitive impairment or dementia [36] (see Supplementary File S3), a history of neurological disease (including brain injury at admission), a history of severe mental illness (including intellectual disability), being non-Spanish speaking, and a life expectancy of <12 months. Patients who were readmitted to the ICU in the month following discharge, those who following the physician’s clinical indications were too ill to participate in follow-up visits because of severe frailty, and those who suffered out-of-hospital neurological complications were also excluded. Patients who did not participate in the one-month follow-up visit were not scheduled for the one-year follow-up visit.

Eligible patients were screened daily by an ICU research nurse and invited to participate when they reached a minimum level of consciousness (Glasgow Coma Scale ≥ 13) and sedation/alertness (Richmond Agitation-Sedation Scale (RASS) −1 to +1). Patients were randomly assigned (ratio 1:1) to the “treatment as usual” (TAU) or “early neurocognitive stimulation” (ENRIC) group using a simple randomization method (blind envelope system) (Figure 1). At enrollment, we obtained written informed consent from patients or their authorized surrogates. If consent was initially obtained from a surrogate (e.g., in case of delirium or deep sedation), we ratified the patient’s consent once they were deemed mentally competent. Patients whose relatives refused to participate, those who did not sign the informed consent form, or those who refused to participate even when their relatives gave prior consent were excluded.

Demographic data, medical comorbidities (Charlson Comorbidity Index, CCI), disease severity (Acute Physiology and Chronic Health Evaluation-II, APACHE-II), and organ failure (Sequential Organ Failure Assessment, SOFA) were collected at admission. Delirium was assessed daily using the Confusion Assessment Method for the ICU (CAM-ICU). Daily doses of opioids and sedatives were also recorded [37]. Sequential data, including delirium and medication, were collected daily from ICU admission until ICU discharge, or for a maximum of 28 days, by experienced ICU clinical and research staff.

During the study, both groups of patients were managed with similar processes of care and only patients in the ENRIC group received adjuvant neurocognitive stimulation every morning in their own beds until ICU discharge when they were alert and calm (RASS −1 to +1). All sessions were guided by a neuropsychologist (therapist) and a virtual avatar (co-therapist) and were supervised by an ICU research nurse or critical care physician. The main objective of the intervention was to provide early neurocognitive stimulation and promote participation in the cognitive exercises, regardless of the accuracy of the responses. The type of cognitive exercises included in each session and their workload were determined daily on the basis of both the patients’ health status [38] and their ability to interact with the ENRIC software (i.e., patients’ alertness) and Kinect^®^ technology (i.e., patients’ ability to raise each arm against gravity). Delirium did not prevent the session from being conducted unless RASS ≥2 (e.g., agitation). The clinical team predefined a session length of 15–20 min, but patients were encouraged to continue as long as they could without fatigue. A detailed description of the protocol of the intervention can be found in the first PoC study [30].

To assess the feasibility of direct outcome measures to detect differences between stimulated vs. non-stimulated groups, we examined PICS-related cognitive and emotional deficits one month and one year after ICU discharge using a comprehensive neuropsychological battery and two mental health questionnaires.

### 2.5. Cognitive and Emotional Assessment

Patients were administered a battery of nine cognitive tests that provided 14 neuropsychological measures [39,40,41,42] and an estimate of intelligence quotient [43]. The raw scores of all neuropsychological measures were converted to z-scores using the normative data set provided by each test and then averaged to create a global neurocognitive score and 6 cognitive indexes: attention, working memory, learning and memory, memory retrieval, executive functions, and processing speed (see Supplementary File S4). These cognitive indexes have already proven effective in detecting cognitive deficits in survivors of critical illness [8].

Patients were also administered 2 mental health questionnaires. The Hospital Anxiety and Depression Scale (HADS) was used to assess non-specific anxiety and depression [44]. The Davidson Trauma Scale (DTS) was used to assess PTSD [45]. The results of these questionnaires can be interpreted dichotomously or continuously. While the HADS Anxiety and Depression scales ranging from 0 to 7 are considered non-pathological, the range of 8 to 21 is pathological. As for the DTS, the non-pathological range is 0 to 39, while the pathological range is 40 to 136. When interpreted on a continuum, higher scores mean more severe symptoms.

### 2.6. Primary and Secondary Outcome Measures

The primary outcome measure was patients’ cognitive functioning and emotional state one month after ICU discharge, measured by the global neurocognitive score, the six cognitive indexes, and the two mental health questionnaires. How these variables changed over a 12 month period was our secondary outcome measure.

### 2.7. Statistical Analysis

Some variables, such as cognitive reserve, were preprocessed prior to statistical analysis (see Supplementary Files S4 and S5). The analysis was performed using SPSS v25. Statistical significance was set at *p* < 0.05. Data normality was checked using skewness, kurtosis, and Shapiro–Wilk tests. Results are presented as means (standard deviation, SD), medians [interquartile range, IQR], or *n* (%). Effect sizes based on Cohen’s *d* (small = 0.2; medium = 0.5; large = 0.8) and partial eta-squared (η_p_^2^; small = 0.01; medium = 0.06; large = 0.14) and 95% confidence intervals are reported for all outcome measures.

The differences between the TAU and ENRIC groups in continuous variables one month after ICU discharge were analyzed using the Student’s *t*-test or the Mann–Whitney *U* test. For categorical variables, the chi-squared (*X*^2^) test was used. To examine how patients’ cognitive and emotional outcomes changed over a 12 month period, we used a general linear model for repeated measures, with factors group (TAU vs. ENRIC), time (one month vs. one year), and group–time interaction.

The possible confounding effect of demographic and clinical variables on patients’ cognitive and emotional outcomes one month after ICU discharge was explored using bivariate regressions. The possible confounders were selected on the basis of their potential to influence cognitive and emotional outcomes according to the previous literature [5,8,14,46]. All variables that reached statistical significance in these screening analyses were included in a multiple linear regression model (one for each cognitive and emotional outcome in which the TAU and ENRIC groups differed). To obtain more consistent models, non-significant variables were excluded step-by-step, starting with the parameters with the highest *p*-value. A final model was constructed including all variables that independently influenced the test score.

## 3. Results

### 3.1. Sample Characteristics

Seventy-two patients were enrolled and randomly assigned to the TAU (*n* = 38) or ENRIC group (*n* = 34). One month after ICU discharge, 21 TAU patients (55.3%) and 21 ENRIC patients (61.8%) completed the first follow-up visit. One year later, 14 TAU patients (66.7%) and 10 ENRIC patients (47.6%) were re-evaluated at a second follow-up visit (Figure 1).

Table 1 summarizes the demographic and clinical characteristics of the patients who completed the first follow-up visit (*n* = 42). No significant differences were observed between the two groups.

Considering only patients who completed both follow-up visits (*n* = 24), we found that ENRIC patients were hospitalized for more days than TAU patients and that, at hospital discharge, the former were mostly transferred to a social-health center (60.0%), whereas the latter were mostly discharged home (85.7%) (see Supplementary File S6).

### 3.2. Characteristics of Early Neurocognitive Stimulation

ENRIC patients received neurocognitive stimulation on at least 2 study days. The first session was administered 9 (IQR: 2–22) days after ICU admission. Sessions were administered on 70.2% (IQR: 22–100%) of the eligible study days until ICU discharge. In total, 106 sessions were administered (mean number of sessions per patient, 5.1 (IQR: 2–14); mean duration of each session, 13.8 (IQR: 5–31) minutes). Patients completed 86.8% (*n* = 92) of the sessions. Reasons for discontinuing a session were fatigue (50.0%), extreme sleepiness (14.3%), dizziness (14.3%), anxiety (14.3%), and confusion (7.1%). No sessions were interrupted early for safety reasons, and no adverse events occurred. Discontinuation did not prevent participation in subsequent sessions. The composition of the first five sessions, with progressively more challenging cognitive exercises, is shown in Supplementary File S7. More details on the safety and tolerability of this intervention can be found in the first PoC study [30].

### 3.3. Cognitive and Emotional Outcomes One Month after ICU Discharge

Table 2 shows that ENRIC patients outperformed TAU patients on the working memory index (*p* = 0.009, *d* = 0.363) but not on the other cognitive outcomes, including global neurocognition, attention, learning and memory, memory retrieval, executive functions, and processing speed.

Regarding emotional state, 16.7% of patients scored within the pathological range for non-specific anxiety (HADS Anxiety: TAU = 21.1% vs. ENRIC = 11.8%, *p* = 0.455, *V* = 0.124), 8.3% for depression (HADS Depression: TAU = 10.5% vs. ENRIC = 5.9%, *p* = 0.615, *V* = 0.084), and 5.7% for PTSD (DTS: TAU = 5.6% vs. ENRIC = 5.9%, *p* = 0.967, *V* = 0.007). For more details, see Table 2 and Supplementary File S8.

### 3.4. Change in Cognitive and Emotional Outcomes over a 12 Month Period

Table 3 summarizes the means, main effects, and interaction effects of group (TAU vs. ENRIC) and time (one month vs. one year) on cognitive and emotional outcomes for patients who completed both follow-up visits (*n* = 24). We only found a main effect of group in the working memory index, with ENRIC patients outperforming TAU patients (*p* = 0.008, η_p_^2^ = 0.282). We did not find any effect of group on emotional outcomes, or any effect of time or group–time interaction on cognitive and emotional outcomes. For more details, see Supplementary File S8.

### 3.5. Impact of Demographic and Clinical Variables on Working Memory Performance

Bivariate regressions (Table 4) show that early neurocognitive stimulation, greater cognitive reserve, and higher dose of morphine equivalents had statistically significant effects on working memory performance one month after ICU discharge. All other variables had *p* > 0.05 and were discarded. In the multiple regression model (Table 4), early neurocognitive stimulation and cognitive reserve, but not the dose of morphine equivalents, remained significant factors. Therefore, the final model only included early neurocognitive stimulation (B = 0.558, 95% CI: 0.15–0.97, *p* = 0.008) and cognitive reserve (B = 0.028, 95% CI: 0.01–0.05, *p* = 0.005) and explained 28.2% of the variance of the working memory index (unadjusted R^2^ = 0.317, F = 9.041, *p* = 0.001). Several properties of interest, such as normal distribution of the residuals, were checked to confirm the goodness of fit of the model.

In view of this result, we re-ran the general linear model of repeated measures for the working memory index adjusting the analysis for cognitive reserve. The results show that the effect of group was maintained (F = 7.105, *p* = 0.014, η_p_^2^ = 0.253), with ENRIC patients outperforming TAU patients. No effect of time (F = 0.000, *p* = 0.997, η_p_^2^ = 0.000) or group-time interaction (F = 0.360, *p* = 0.555, η_p_^2^ = 0.017) was found. Cognitive reserve was significant for working memory performance (F = 8.298, *p* = 0.009, η_p_^2^ = 0.283).

## 4. Discussion

We have demonstrated for the first time that non-immersive VR-based neurocognitive interventions can be useful solutions to mitigate certain PICS-related sequelae in critically ill patients undergoing MV. Specifically, we found that patients who received neurocognitive stimulation in the ICU had better outcomes in the working memory domain one month and one year after discharge compared to patients who received only standard ICU care. This result suggests two preliminary conclusions: first, that these types of digital therapies could have a positive impact on certain short-term cognitive outcomes in survivors of critical illness and, second, that their benefits could persist over time.

Deep sedation is one of the main barriers to early physical and occupational therapy in the ICU [47]. In our study, neurocognitive stimulation began when patients reached RASS −1 to +1 and was successfully applied during 70.2% of their time in the ICU, reflecting a wide therapeutic window. Only 13.2% of sessions were interrupted due to sudden patient indisposition, in no case related to safety reasons or adverse events (e.g., inadvertent removal of catheters or endotracheal tubes). Therefore, the present findings are consistent with those of the first PoC study in which we already demonstrated that ENRIC therapy is a safe and well-tolerated intervention [30]. However, the mean duration of the sessions [13.8 min] was shorter than expected [15–20 min], suggesting that sessions lasting 12–15 min may be more appropriate than longer ones in critically ill patients, as is also the case in a recently published study in stroke patients [48].

One of the main findings of this study is that ENRIC therapy may help improve working memory outcomes in survivors of critical illness. The neural substrate of working memory primarily involves the dorsolateral prefrontal cortex [49]. This is in line with previous findings from our group, using heart rate variability as a surrogate outcome measure, in which we already demonstrated that ENRIC therapy effectively stimulates the prefrontal regions of the brain [30]. Indeed, working memory is supported by a broader network of fronto-parietal brain regions that provide the temporary storage and manipulation of information necessary for the proper functioning of other cognitive domains, such as language understanding, learning, and reasoning [50]. Therefore, although our results do not reveal any direct impact of ENRIC therapy beyond the domain of working memory, it is conceivable that the better performance observed in this cognitive function positively influences general cognition and, in particular, memory and executive functions. Nevertheless, whether this effect is transferred to *real world* skills is a controversial issue that needs to be explored further [51].

Brummel et al. [21] were the first to evaluate the feasibility and safety of a combined intervention of early physical therapy and neurocognitive stimulation. However, they found no difference between the control and the experimental groups in cognitive outcomes three months after ICU discharge. The use of classic paper-and-pencil neuropsychological exercises in the intervention of Brummel et al. may help explain their different results in comparison with those of ENRIC therapy. It is possible that VR environments and Kinect^®^ technology offer advantages in terms of motivation and engagement with neurocognitive stimulation. Another explanation could be that we have used a broader neuropsychological battery than the one used in the study by Brummel et al., allowing us to detect subtler differences in cognitive outcomes [8].

Cognitive reserve has been identified as a protective factor against cognitive decline in several studies in patients with neurocognitive and mental health disorders [52,53,54]. In our study, along with the significant impact of early neurocognitive stimulation, greater cognitive reserve was also associated with better working memory outcomes in survivors of critical illness. In contrast, we found no impact of age, gender, and duration of MV [55,56]. Patients in our study had a shorter duration of delirium than patients in other cohorts [6,10]; perhaps for this reason we found no relationship between this variable and cognitive performance.

Unlike other studies that found reduced levels of anxiety and depression following VR-based emotional interventions [26,27,28,29], we found no significant differences in emotional outcomes between the stimulated and non-stimulated groups. However, at one-month follow-up, 50% fewer patients in the ENRIC group experienced pathological levels of non-specific anxiety (11.8% vs. 21.1%) and depression (5.9% vs. 10.5%) compared with patients who received only standard ICU care. This difference, although not statistically significant, is clinically relevant and should be borne in mind. Surprisingly, patients who received early neurocognitive stimulation showed a trend towards a worsening of depressive symptoms at one-year follow-up. The longer hospital stay and discharge destination in this subgroup of patients, both probably related to a slower functional recovery or to other variables not considered in our study (e.g., ICU acquired weakness), may help explain this result [5,14].

In our study, the one-year follow-up is limited to a small number of patients at a single center, and therefore the results related to these data should be interpreted with caution. It is also worth mentioning that the research staff was not blinded to group assignment and that the same neuropsychologists who administered the neurocognitive stimulation also assessed its impact on cognitive and emotional outcomes at the two follow-up visits. However, our results are strengthened by the study’s RCT design, the inclusion of both medical and surgical patients, and the daily monitoring of delirium and medication. Moreover, the wide therapeutic window of the intervention, its specific design and tailoring to the ICU environment and MV patient, and the use of a comprehensive neuropsychological battery all add value to our results. Nevertheless, the use of a broader psychopathological assessment might have increased our sensitivity to detect subtler differences in patients’ emotional state. Other issues that should be addressed in future trials are the personalization of the intervention to the patient’s health status (including the frequency of the sessions and the type, duration, and difficulty of cognitive exercises) and its impact on functionality and quality of life.

In line with the challenges and perspectives in the field of e-health and m-health, our results support the introduction of new technologies based on the principles of cognitive rehabilitation as a complement to standard ICU care. These novel interventions should always focus on personalized management and patient-centered decision making, empowering patients to be active players in their recovery process.

## 5. Conclusions

Non-immersive VR-based early neurocognitive interventions can be a useful solution to improve short- and long-term working memory outcomes in ICU survivors. Further studies with larger samples are now needed to corroborate these results in a definitive efficacy trial. If confirmed, ENRIC therapy and other VR-based technological tools may become cost-effective solutions for hospitals and, in particular, ICUs, as they could help reduce the medical and financial costs associated with PICS. The present findings can foster the introduction of digital therapies as adjuvant, feasible, and safe interventional tools in the ICU. This is of particular interest in the context of the paradigm shift from face-to-face healthcare to telemedicine and telerehabilitation, as well as in the adoption of new technological environments highly adaptable to patients’ needs.

## Figures and Tables

**Table 1:** Demographic and clinical characteristics of patients evaluated one month after ICU discharge. Mean (SD) or Median [IQR] are reported, unless otherwise specified.

	All Patients	TAU Group	ENRIC Group	*p*
*n*	42	21	21	
Age, years	68.8 [35.7–85.9]	67.7 [36.6–85.3]	69.1 [35.7–85.9]	0.414 ^b^
Education, years	7.9 (4.6)	7.7 (4.9)	8.1 (4.5)	0.767 ^a^
Female gender, *n* (%)	25 (59.5)	12 (57.1)	13 (61.9)	0.753 ^c^
Cognitive reserve, standard score	98.5 (11.1)	97.7 (11.7)	99.3 (10.7)	0.646 ^a^
Diagnosis, *n* (%)				0.779 ^c^
Medical	24 (57.1)	11 (52.4)	13 (61.9)	
Acute respiratory failure	10	5	5	
Septic shock	6	4	2	
Pneumonia	4	1	3	
Pancreatitis	3	1	2	
Toxic intake	1	0	1	
Unplanned surgery	12 (28.6)	7 (33.3)	5 (23.8)	
Peritonitis	3	0	3	
Multiple trauma	3	1	2	
Abdominal aortic aneurism	3	3	0	
Intestinal perforation	1	1	0	
Intestinal ischemia	1	1	0	
Esophageal perforation	1	1	0	
Planned surgery	6 (14.3)	3 (14.3)	3 (14.3)	
Hemorrhagic shock	2	0	2	
Tumor	4	3	1	
CCI	4 [0–8]	3 [0–8]	4 [0–7]	0.148 ^b^
APACHE-II	20.9 (7.7)	20.2 (7.2)	21.7 (8.4)	0.542 ^a^
SOFA at admission	8.3 (3.8)	7.4 (3.6)	9.1 (3.8)	0.131 ^a^
Length of ICU stay, days	13 [5–76]	10 [5–73]	16 [6–76]	0.252 ^b^
Length of hospital stay, days	28 [7–169]	19 [9–169]	28 [7–103]	0.588 ^b^
Destination at hospital discharge, *n* (%)				0.240 ^c^
Home	29 (69.0)	15 (71.4)	14 (66.7)	
Home hospitalization	2 (4.8)	2 (9.5)	0 (0.0)	
Social-health center	11 (26.2)	4 (19.1)	7 (33.3)	
Duration of MV, days	7 [2–71]	7 [2–51]	8 [3–71]	0.331 ^b^
Duration of delirium, days	0.5 [0–8]	1 [0–8]	0 [0–6]	0.655 ^b^
ARDS, *n* (%)	2 (4.8)	0 (0.0)	2 (9.5)	0.147 ^c^
Septic shock, *n* (%)	17 (40.5)	7 (33.3)	10 (47.6)	0.346 ^c^
Cardiac arrest, *n* (%)	1 (2.4)	0 (0.0)	1 (4.8)	0.311 ^c^
Morphine equivalents (mg/kg/day)	1.6 [0.1–12.2]	1.6 [0.1–8.4]	1.9 [0.1–12.2]	0.498 ^b^
Midazolam equivalents (mg/kg/day)	3.8 [0.1–77.1]	3.2 [0.2–77.1]	5.3 [0.1–37.8]	0.361 ^b^

ICU—intensive care unit, SD—standard deviation, IQR—interquartile range, TAU—treatment as usual, ENRIC—early neurocognitive stimulation, CCI—charlson comorbidity index, APACHE-II—acute physiology and chronic health evaluation-II, SOFA—sequential organ failure assessment, MV—mechanical ventilation, ARDS—acute respiratory distress syndrome. Standard score: mean ± SD = 100 ± 15. ^a^ Student’s *t*-test, ^b^ Mann–Whitney *U* test, ^c^ chi-squared test. No significant differences at *p* < 0.05.

**Table 2:** Cognitive and emotional outcomes one month after ICU discharge (Student’s *t*-test). Mean (SD) is reported.

	TAU (*n* = 21)	ENRIC (*n* = 21)	*t*	*p*	95% CI	*d*
Cognitive outcomes, z-score						
Attention	−0.06 (0.68)	0.26 (0.74)	−1.453	0.154	−0.76 to 0.12	0.190
Working memory **	−0.17 (0.60)	0.44 (0.81)	−2.741	0.009	−1.05 to −0.16	0.363
Learning and memory	−0.86 (1.23)	−0.90 (0.91)	0.137	0.891	−0.63 to 0.72	0.019
Memory retrieval	−1.03 (1.49)	−0.57 (0.94)	−1.223	0.230	−1.24 to 0.31	0.209
Executive functions	−1.06 (1.05)	−1.11 (1.09)	0.146	0.764	−0.62 to 0.72	0.024
Processing speed	−0.99 (1.00)	−0.77 (1.35)	−0.579	0.566	−0.96 to 0.53	0.102
Global neurocognition	−0.78 (0.79)	−0.57 (0.81)	−0.852	0.339	−0.71 to 0.29	0.118
Emotional outcomes, raw score						
HADS Anxiety	4.00 (4.16)	3.00 (4.56)	0.021	0.983	−0.28 to 0.28	0.240
HADS Depression	2.79 (2.96)	3.29 (3.92)	−1.128	0.270	−0.40 to 0.12	0.135
Davidson Trauma Scale	11.94 (12.32)	8.94 (21.91)	1.075	0.292	−0.17 to 0.52	0.363

ICU—intensive care unit, SD—standard deviation, TAU—treatment as usual, ENRIC—early neurocognitive stimulation, HADS—hospital anxiety and depression scale. Z-score: mean ± SD = 0 ± 1. ** *p* < 0.01.

**Table 3:** Change in cognitive and emotional outcomes over a 12 month period (general linear model of repeated measures). Mean (SD) is reported.

	TAU (*n* = 14)		ENRIC (*n* = 10)		Group			Time			Group–Time		
	One Month	One Year	One Month	One Year	F	*p*	η_p_^2^	F	*p*	η_p_^2^	F	*p*	η_p_^2^
Cognitive outcomes, z-score													
Attention	0.09 (0.75)	−0.04 (0.75)	0.49 (0.75)	0.33 (1.10)	1.389	0.251	0.059	1.340	0.259	0.057	0.020	0.889	0.001
Working memory **	−0.07 (0.62)	0.01 (0.65)	0.88 (0.88)	0.81 (0.98)	8.661	0.008	0.282	0.000	0.998	0.000	0.410	0.528	0.018
Learning and memory	−0.95 (1.22)	−1.02 (1.14)	−0.80 (0.99)	−1.00 (1.39)	0.037	0.850	0.002	0.447	0.511	0.020	0.111	0.742	0.005
Memory retrieval	−0.85 (1.15)	−0.92 (1.11)	−0.30 (1.00)	−0.84 (1.33)	0.562	0.461	0.025	1.798	0.194	0.076	1.041	0.319	0.045
Executive functions	−0.95 (1.09)	−1.22 (1.60)	−1.12 (1.31)	−1.54 (1.38)	0.239	0.630	0.011	1.979	0.173	0.083	0.096	0.760	0.004
Processing speed	−1.06 (1.11)	−0.88 (1.35)	−0.02 (0.84)	−0.28 (0.99)	2.961	0.101	0.129	0.067	0.799	0.003	2.226	0.151	0.100
Global neurocognition	−0.71 (0.77)	−0.73 (0.88)	−0.27 (0.84)	−0.47 (0.87)	1.164	0.292	0.050	0.869	0.361	0.038	0.504	0.485	0.022
Emotional outcomes, raw score													
HADS Anxiety	4.17 (4.90)	3.75 (4.73)	4.78 (5.65)	4.56 (3.05)	0.604	0.450	0.041	0.507	0.488	0.035	0.294	0.596	0.021
HADS Depression	2.58 (2.91)	2.75 (3.08)	4.78 (4.66)	6.22 (5.22)	0.371	0.082	0.030	0.553	0.471	0.044	0.403	0.538	0.032
Davidson Trauma Scale	11.92 (14.25)	7.75 (10.02)	10.78 (17.00)	7.00 (9.12)	0.922	0.632	0.093	0.235	0.640	0.025	4.278	0.949	0.322

SD—standard deviation, TAU—treatment as usual, ENRIC—early neurocognition stimulation, HADS—hospital anxiety and depression scale. Z-score: mean ± SD = 0 ± 1. ** Significant main effect of group (*p* < 0.01).

**Table 4:** Influence of demographic and clinical variables on working memory performance one month after ICU discharge.

	Bivariate Analyses		Multiple Linear Regression	
	B (95% CI)	*p*	B (95% CI)	*p*
Group *	0.602 (0.16 to 1.05)	0.009	0.464 (0.05 to 0.88)	0.029
Age	−0.002 (−0.02 to 0.02)	0.809		
Gender	−0.147 (−0.64 to 0.34)	0.548		
Cognitive reserve *	0.030 (0.01 to 0.05)	0.005	0.026 (0.01 to 0.05)	0.009
Diagnosis	0.101 (−0.17 to 0.37)	0.458		
CCI	−0.065 (−0.17 to 0.04)	0.206		
APACHE-II	−0.006 (−0.04 to 0.03)	0.687		
Length of ICU stay	0.005 (−0.01 to 0.02)	0.528		
Length of hospital stay	0.003 (−0.01 to 0.01)	0.478		
Duration of MV	0.007 (−0.01 to 0.02)	0.393		
Duration of delirium	−0.036 (−0.15 to 0.08)	0.520		
Morphine equivalents	0.097 (0.02 to 0.18)	0.019	0.058 (−0.02 to 0.13)	0.117
Midazolam equivalents	0.008 (−0.01 to 0.03)	0.392		

ICU—intensive care unit, TAU—treatment as usual, ENRIC—early neurocognitive stimulation, CCI—charlson comorbidity index, APACHE-II—acute physiology and chronic health evaluation II, MV—mechanical ventilation. * *p* < 0.05 in the multiple linear regression model.

## Data Availability

The data presented in this study are available on request from the corresponding author. The data are not publicly available due to privacy restrictions.

## Supplementary Materials

The following are available online at https://www.mdpi.com/article/10.3390/jpm11121260/s1, File S1: ENRIC Technology Features, File S2: ENRIC Platform Video, File S3: Participants and Procedure, File S4: Cognitive Assessment, File S5: Data Preprocessing, File S6: Sample Characteristics, File S7: Characteristics of Early Neurocognitive Stimulation, File S8: Cognitive and Emotional Outcomes.
