# Supplementary material for: Virtual Reality-Based Early Neurocognitive Stimulation in Critically Ill Patients: A Pilot Randomized Clinical Trial
Source: J Pers Med. 2021 Nov 29;11(12):1260. doi: 10.3390/jpm11121260 (PMC8703623; doi:10.3390/jpm11121260)
Supplement: Supplementary file 1 [file jpm-11-01260-s001.zip › Supplementary File S1.pdf]

Supplementary File S1: ENRIC Technology Features

Article title: **Virtual reality-based early neurocognitive stimulation in critically ill patients: A pilot randomized clinical trial**

Authors: Guillem Navarra-Ventura, Gemma Gomà, Candelaria de Haro, Mercè Jodar, Leonardo Sarlabous, David Hernando, Raquel Bailón, Ana Ochagavía, Lluís Blanch, Josefina López-Aguilar* and Sol Fernández-Gonzalo*

* Co-senior authors

Corresponding author: Guillem Navarra-Ventura (gnavarra@tauli.cat)

Critical Care Center, Hospital Universitari Parc Taulí, Institut d'Investigació i Innovació Parc Taulí I3PT, Universitat Autònoma de Barcelona, 08208 Sabadell, Catalonia (Spain). Tel. +34 937 23 66 73

Journal name: Journal of Personalized Medicine

Methods

ENRIC Technology Features

The Early Neurocognitive Rehabilitation in Intensive Care (ENRIC) platform consists of a central processing unit, a flat-screen TV, and a Kinect® motion sensor (Microsoft®, Washington) placed on a medical cart for easy transfer to each intensive care unit (ICU) bed. The central processing unit captures and interprets patient movements through smart middleware connecting the neurocognitive stimulation software with input from the motion sensor (Figure S1). Therefore, patients do not need to directly touch any physical device (e.g., mouse, keyboard, joystick, etc.), which substantially minimizes the risk of cross-infection. The ENRIC platform is compatible with software that collects and stores physiological data from bedside monitors and ventilators (Better Care Link®, BetterCare SL, Barcelona) [1], which was used to adapt the cognitive exercise workload to the patient's daily medical condition.

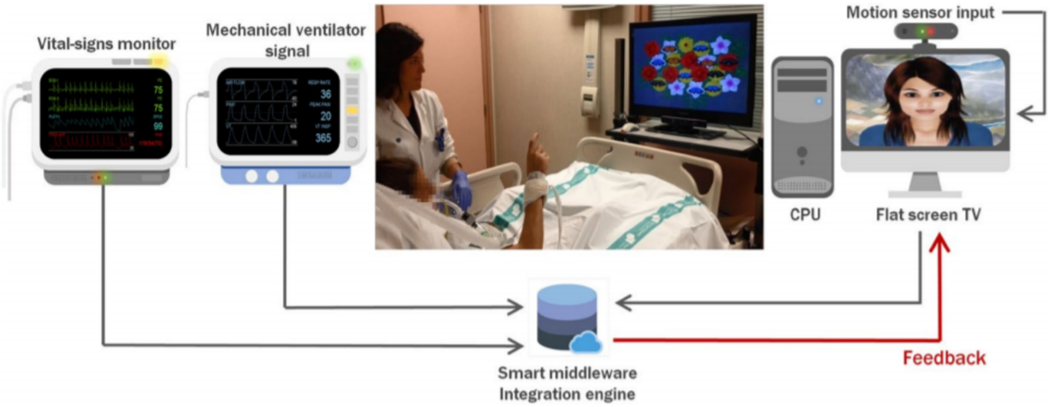

Figure S1. Schematic diagram of the Early Neurocognitive Rehabilitation in Intensive Care (ENRIC) platform (reproduced with permission [2]).

References

1. Blanch L, Sales B, Montanya J et al (2012) Validation of the Better Care® system to detect ineffective efforts during expiration in mechanically ventilated patients: a pilot study. Intensive Care Med 38:772–780
2. Turon M, Fernandez-Gonzalo S, Jodar M, et al (2017) Feasibility and safety of virtual-reality-based early neurocognitive stimulation in critically ill patients. Ann Intensive Care 7:81
