# Supplementary material for: Virtual Reality-Based Early Neurocognitive Stimulation in Critically Ill Patients: A Pilot Randomized Clinical Trial
Source: J Pers Med. 2021 Nov 29;11(12):1260. doi: 10.3390/jpm11121260 (PMC8703623; doi:10.3390/jpm11121260)
Supplement: Supplementary file 1 [file jpm-11-01260-s001.zip › Supplementary File S3.pdf]

Supplementary File S3: Participants and Procedure

Article title: **Virtual reality-based early neurocognitive stimulation in critically ill patients: A pilot randomized clinical trial**

Authors: Guillem Navarra-Ventura, Gemma Gomà, Candelaria de Haro, Mercè Jodar, Leonardo Sarlabous, David Hernando, Raquel Bailón, Ana Ochagavía, Lluís Blanch, Josefina López-Aguilar*, & Sol Fernández-Gonzalo*

* Co-senior authors

Corresponding author: Guillem Navarra-Ventura (gnavarra@tauli.cat)

Critical Care Center, Hospital Universitari Parc Taulí, Institut d'Investigació i Innovació Parc Taulí I3PT, Universitat Autònoma de Barcelona, 08208 Sabadell, Catalonia (Spain). Tel. +34 937 23 66 73

Journal name: Journal of Personalized Medicine

Methods

Participants and Procedure

Previous cognitive impairment or dementia was checked in the patients' clinical records and by interviewing the relatives. In patients aged ≥60, pre-existing cognitive impairment was recorded using the short form of the Spanish version of the Informant Questionnaire on Cognitive Decline in the Elderly (IQCODE) [1] filled in by relatives. Patients with scores ≥3.56 were excluded as they were at risk of probable cognitive impairment.

References

1. Jorm AF (2004) The Informant Questionnaire on Cognitive Decline in the Elderly (IQCODE): a review. Int Psychogeriatrics 16:275–293
