# Supplementary material for: Virtual Reality-Based Early Neurocognitive Stimulation in Critically Ill Patients: A Pilot Randomized Clinical Trial
Source: J Pers Med. 2021 Nov 29;11(12):1260. doi: 10.3390/jpm11121260 (PMC8703623; doi:10.3390/jpm11121260)
Supplement: Supplementary file 1 [file jpm-11-01260-s001.zip › Supplementary File S4.pdf]

Supplementary File S4: Cognitive Assessment

Article title: **Virtual reality-based early neurocognitive stimulation in critically ill patients: A pilot randomized clinical trial**

Authors: Guillem Navarra-Ventura, Gemma Gomà, Candelaria de Haro, Mercè Jodar, Leonardo Sarlabous, David Hernando, Raquel Bailón, Ana Ochagavía, Lluís Blanch, Josefina López-Aguilar*, & Sol Fernández-Gonzalo*

* Co-senior authors

Corresponding author: Guillem Navarra-Ventura (gnavarra@tauli.cat)

Critical Care Center, Hospital Universitari Parc Taulí, Institut d'Investigació i Innovació Parc Taulí I3PT, Universitat Autònoma de Barcelona, 08208 Sabadell, Catalonia (Spain). Tel. +34 937 23 66 73

Journal name: Journal of Personalized Medicine

Methods

Cognitive Assessment

Table S1 shows the neuropsychological tests that compose the global neurocognitive score and the different cognitive indexes, as well as the formulas for calculating them.

Table S1 Neuropsychological tests that compose each cognitive index

Cognitive Index	Neuropsychological Tests and Formulas for Calculating the Cognitive Indexes
Attention	(WAIS-III forward digit span + WMS-III forward spatial span)/2
Working memory	(WAIS-III backward digit span + WMS-III backward spatial span)/2
Learning and memory	(RAVLT learning slope (trials 1-5) + RAVLT delayed recall (30 minutes)/2
Memory retrieval	(RAVLT recognition + Benton Visual Retention Test form M)/2
Executive functions	(Trail Making Test part B + Stroop test Color-Word + FAS)/3
Processing speed	(WAIS-III symbol search + Trail Making Test part A + Stroop test Word-Reading)/3
Global neurocognition	(Sum of all neuropsychological tests)/14

WAIS-III Wechsler adult intelligence scale, third edition, *WMS-III* Wechsler memory scale, third edition, *RAVLT* Rey auditory verbal learning test, *FAS* Phonemic verbal fluency. Raw data were corrected for age, education and, in some cases, gender, based on the normative datasets provided by each test [1–4].

References

1. Wechsler D (1999) Wechsler Adult Intelligence Scale, 3rd edition: WAIS-III. TEA Ediciones, Barcelona, Catalonia (Spain)
2. Wechsler D (1997) Wechsler Memory Scale, 3rd edition: WMS-III. TEA Ediciones, Barcelona, Catalonia (Spain)
3. Strauss E, Sherman EMS, Spreen O (2006) A compendium of neuropsychological tests: Administration, norms, and commentary, 3rd edition. Oxford University Press, New York, NY
4. Lezak MD, Howieson DB, Bigler ED et al (2012) Neuropsychological Assessment, 5th edition. Oxford University Press, New York, NY
