# Supplementary material for: Virtual Reality-Based Early Neurocognitive Stimulation in Critically Ill Patients: A Pilot Randomized Clinical Trial
Source: J Pers Med. 2021 Nov 29;11(12):1260. doi: 10.3390/jpm11121260 (PMC8703623; doi:10.3390/jpm11121260)
Supplement: Supplementary file 1 [file jpm-11-01260-s001.zip › Supplementary File S5.pdf]

Supplementary File S5: Data Preprocessing

Article title: **Virtual reality-based early neurocognitive stimulation in critically ill patients: A pilot randomized clinical trial**

Authors: Guillem Navarra-Ventura, Gemma Gomà, Candelaria de Haro, Mercè Jodar, Leonardo Sarlabous, David Hernando, Raquel Bailón, Ana Ochagavía, Lluís Blanch, Josefina López-Aguilar*, and Sol Fernández-Gonzalo*

* Co-senior authors

Corresponding author: Guillem Navarra-Ventura (gnavarra@tauli.cat)

Critical Care Center, Hospital Universitari Parc Taulí, Institut d'Investigació i Innovació Parc Taulí I3PT, Universitat Autònoma de Barcelona, 08208 Sabadell, Catalonia (Spain). Tel. +34 937 23 66 73

Journal name: Journal of Personalized Medicine

Methods

Data Preprocessing

In addition to calculating the six cognitive indexes and the global neurocognitive score [Supplementary File S4], the variable "diagnosis" was operationalized in three categories: medical, unplanned surgery and planned surgery. Furthermore, the accumulated doses of opioids (morphine and fentanyl) and sedatives (midazolam and propofol) during the intensive care unit (ICU) stay were converted to morphine and midazolam equivalents, respectively [1]. Finally, educational attainment (number of full-time years in formal education) and literacy (National Adult Reading Test score, NART [2]) were adopted as our two proxies of cognitive reserve. To obtain the "cognitive reserve" variable, we first transformed the variable "years of education" to standard scores using the mean and standard deviation of the entire sample and then averaged this score with the estimated intelligence quotient (NART) [3].

References

1. de Haro C, Magrans R, López-Aguilar J et al (2019) Effects of sedatives and opioids on trigger and cycling asynchronies throughout mechanical ventilation: an observational study in a large dataset from critically ill patients. Crit Care 23:245
2. Gomar JJ, Ortiz-Gil J, McKenna PJ et al (2011) Validation of the Word Accentuation Test (TAP) as a means of estimating premorbid IQ in Spanish speakers. Schizophr Res 128:175–176
3. Fernández-Gonzalo S, Navarra-Ventura G, Bacardit N et al (2020) Cognitive phenotypes 1 month after ICU discharge in mechanically ventilated patients: a prospective observational cohort study. Crit Care 24:618
