# Supplementary material for: Virtual Reality-Based Early Neurocognitive Stimulation in Critically Ill Patients: A Pilot Randomized Clinical Trial
Source: J Pers Med. 2021 Nov 29;11(12):1260. doi: 10.3390/jpm11121260 (PMC8703623; doi:10.3390/jpm11121260)
Supplement: Supplementary file 1 [file jpm-11-01260-s001.zip › Supplementary File S6.pdf]

Supplementary File S6: Sample Characteristics

Article title: **Virtual reality-based early neurocognitive stimulation in critically ill patients: A pilot randomized clinical trial**

Authors: Guillem Navarra-Ventura, Gemma Gomà, Candelaria de Haro, Mercè Jodar, Leonardo Sarlabous, David Hernando, Raquel Bailón, Ana Ochagavía, Lluís Blanch, Josefina López-Aguilar*, & Sol Fernández-Gonzalo*

* Co-senior authors

Corresponding author: Guillem Navarra-Ventura (gnavarra@tauli.cat)

Critical Care Center, Hospital Universitari Parc Taulí, Institut d'Investigació i Innovació Parc Taulí I3PT, Universitat Autònoma de Barcelona, 08208 Sabadell, Catalonia (Spain). Tel. +34 937 23 66 73

Journal name: Journal of Personalized Medicine

Results

Sample Characteristics

Table S2 shows the demographic and clinical characteristics of patients who completed both follow-up visits.

Table S2 Characteristics of patients evaluated one month and one year after ICU discharge. Mean (SD) or Median [IQR] are reported unless otherwise specified

	All Patients	TAU Group	ENRIC Group	*p*
n	24	14	10	
Age, years	63.9 (11.7)	64.0 (11.1)	63.7 (13.20)	0.957 [a]
Education, years	8.8 (3.7)	8.2 (3.2)	9.5 (4.3)	0.407 [a]
Female gender, *n* (%)	11 (45.8)	7 (50.0)	4 (40.0)	0.628 [c]
Cognitive reserve, standard score	100.7 (8.3)	99.2 (6.6)	102.9 (10.3)	0.293 [a]
Diagnosis, *n* (%)				0.640 [c]
Medical	16 (66.7)	10 (71.5)	6 (60.0)	
Acute respiratory failure	7	5	2	
Septic shock	5	4	1	
Pneumonia	3	1	2	
Pancreatitis	1	0	1	
Unplanned surgery	5 (20.8)	3 (21.4)	2 (20.0)	
Peritonitis	2	0	1	
Multiple trauma	2	1	1	
Abdominal aortic aneurism	1	2	0	
Planned surgery	3 (12.5)	1 (7.1)	2 (20.0)	
Hemorrhagic shock	2	0	2	
Tumor	1	1	0	
CCI	2.5 [0-8]	2.5 [0-8]	2.5 [0-6]	0.789 [b]
APACHE-II	20.0 (8.3)	21.4 (7.7)	18.0 (9.1)	0.342 [a]
SOFA at admission	8.1 (3.5)	8.1 (3.4)	8.1 (4.0)	0.985 [a]
Length of ICU stay, days	16.5 [5-73]	9.5 [5-73]	20.5 [6-42]	0.151 [b]
Length of hospital stay, days *	31.5 [9-169]	19 [9-169]	43 [19-103]	0.043 [b]
Destination at hospital discharge, *n* (%) *				0.048 [c]
Home	14 (58.3)	10 (71.4)	4 (40.0)	
Home hospitalization	2 (8.3)	2 (14.3)	0 (0.0)	
Social-health center	8 (33.3)	2 (14.3)	6 (60.0)	
Duration of MV, days	8 [2-51]	7 [2-51]	13 [3-38]	0.083 [b]
Duration of delirium, days	0.5 [0-7]	0.5 [0-7]	0.5 [0-6]	0.729 [b]
ARDS, *n* (%)	2 (8.3)	0 (0.0)	2 (20.0)	0.081 [c]
Septic shock, *n* (%)	10 (41.7)	5 (35.7)	5 (50.0)	0.484 [c]
Cardiac arrest, *n* (%)	0 (0.0)	0 (0.0)	0 (0.0)	N/A
Morphine equivalents (mg/kg/day)	1.8 [0.1-12.2]	1.6 [0.1-8.4]	4.5 [0.3-12.2]	0.089 [b]
Midazolam equivalents (mg/kg/day)	4.5 [0.2-37.8]	3.2 [0.2-31.9]	8.1 [0.3-37.8]	0.072 [b]

SD Standard deviation, *IQR* Interquartile range, *ICU* Intensive care unit, *TAU* Treatment as usual, *ENRIC* Early neurocognitive stimulation, *CCI* Charlson comorbidity index, *APACHE-II* Acute physiology and chronic health evaluation-II, *SOFA* Sequential organ failure assessment, *MV* Mechanical ventilation, *ARDS* Acute respiratory distress syndrome, *N/A* does not apply. Standard score: mean ± SD = 100 ± 15. [a] Student's t-test, [b] Mann-Whitney U test, [c] Chi-square test. * $p < 0.05$.
