# Supplementary material for: Virtual Reality-Based Early Neurocognitive Stimulation in Critically Ill Patients: A Pilot Randomized Clinical Trial
Source: J Pers Med. 2021 Nov 29;11(12):1260. doi: 10.3390/jpm11121260 (PMC8703623; doi:10.3390/jpm11121260)
Supplement: Supplementary file 1 [file jpm-11-01260-s001.zip › Supplementary File S7.pdf]

Supplementary File S7: Characteristics of Early Neurocognitive Stimulation

Article title: **Virtual reality-based early neurocognitive stimulation in critically ill patients: A pilot randomized clinical trial**

Authors: Guillem Navarra-Ventura, Gemma Gomà, Candelaria de Haro, Mercè Jodar, Leonardo Sarlabous, David Hernando, Raquel Bailón, Ana Ochagavía, Lluís Blanch, Josefina López-Aguilar*, & Sol Fernández-Gonzalo*

* Co-senior authors

Corresponding author: Guillem Navarra-Ventura (gnavarra@tauli.cat)

Critical Care Center, Hospital Universitari Parc Taulí, Institut d'Investigació i Innovació Parc Taulí I3PT, Universitat Autònoma de Barcelona, 08208 Sabadell, Catalonia (Spain). Tel. +34 937 23 66 73

Journal name: Journal of Personalized Medicine

Results

Characteristics of Early Neurocognitive Stimulation

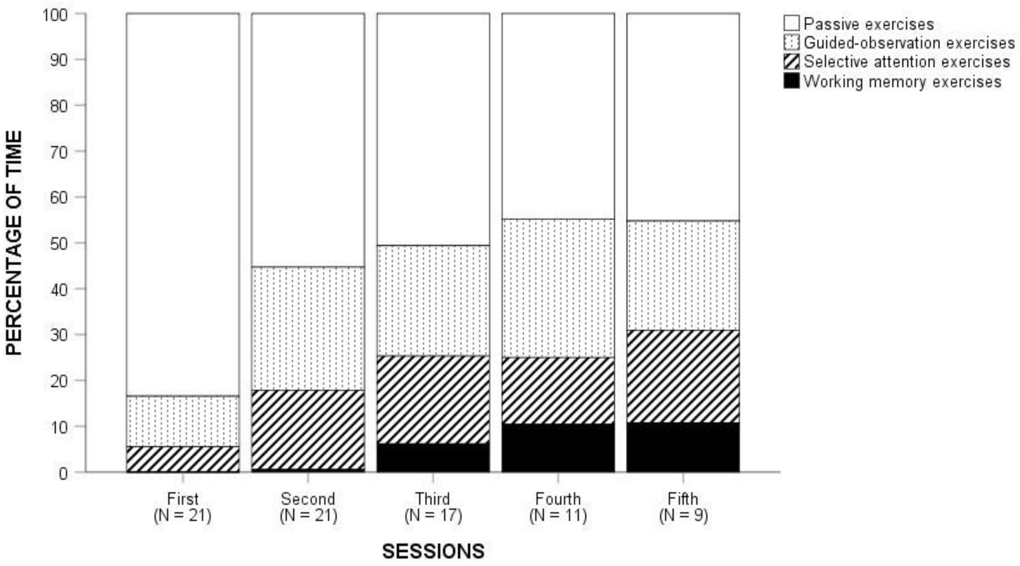

Figure S2. Time distribution (%) of cognitive exercises for each session during the first five sessions. In the first session, *passive exercises* requiring simple attention and gross motor functions were the most performed exercises. In the following sessions, the time spent on *passive exercises* gradually decreased, while the time spent on *guided-observation exercises* and exercises focusing on *selective attention* and *working memory* progressively increased. The more complex exercises focused on *working memory* were performed mainly from the third session onwards.
