# Supplementary material for: Virtual Reality-Based Early Neurocognitive Stimulation in Critically Ill Patients: A Pilot Randomized Clinical Trial
Source: J Pers Med. 2021 Nov 29;11(12):1260. doi: 10.3390/jpm11121260 (PMC8703623; doi:10.3390/jpm11121260)
Supplement: Supplementary file 1 [file jpm-11-01260-s001.zip › Supplementary File S8.pdf]

Supplementary File S8: Cognitive and Emotional Outcomes

Article title: **Virtual reality-based early neurocognitive stimulation in critically ill patients: A pilot randomized clinical trial**

Authors: Guillem Navarra-Ventura, Gemma Gomà, Candelaria de Haro, Mercè Jodar, Leonardo Sarlabous, David Hernando, Raquel Bailón, Ana Ochagavía, Lluís Blanch, Josefina López-Aguilar*, & Sol Fernández-Gonzalo*

* Co-senior authors

Corresponding author: Guillem Navarra-Ventura (gnavarra@tauli.cat)

Critical Care Center, Hospital Universitari Parc Taulí, Institut d'Investigació i Innovació Parc Taulí I3PT, Universitat Autònoma de Barcelona, 08208 Sabadell, Catalonia (Spain). Tel. +34 937 23 66 73

Journal name: Journal of Personalized Medicine

Results

Cognitive Outcomes One Month after ICU Discharge

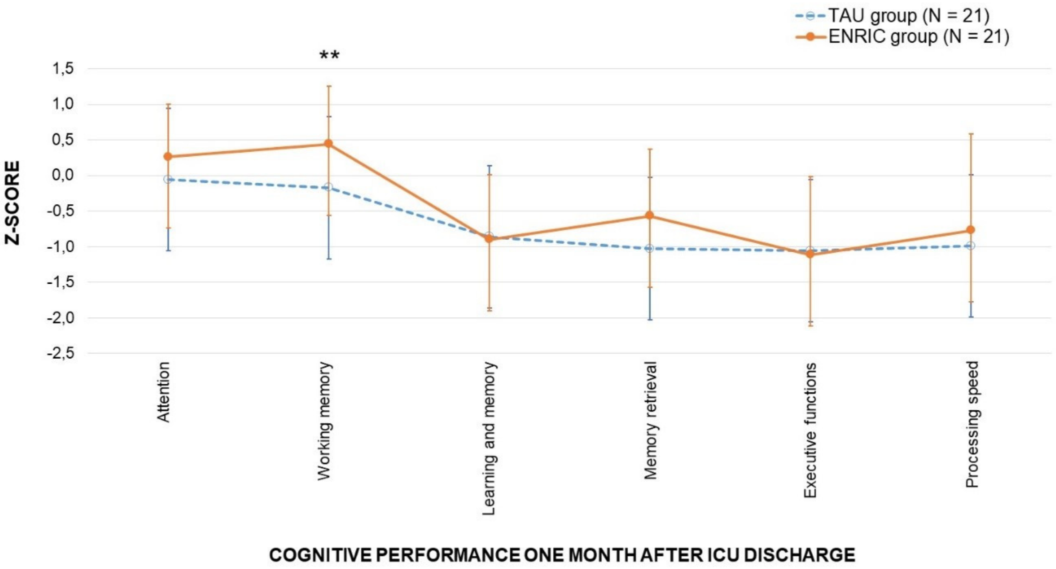

Figure S3. Cognitive outcomes 1 month after ICU discharge [mean (SD)]. ** $p < 0.01$.

Cognitive and Emotional Outcomes One Year after ICU Discharge

Table S3 shows that ENRIC patients performed significantly better than TAU patients on the working memory index, but not on the other cognitive and emotional outcomes.

Table S3. Cognitive and emotional outcomes one year after ICU discharge (student's *t*-test).

	**TAU Group (*n* = 14)**	**ENRIC Group (*n* = 10)**	***t***	***p***	**95% CI**	**Cohen's *d***
***Cognitive outcomes**, z-score (mean, SD)*						
Attention	-0.04 (0.75)	0.33 (1.10)	-0.960	0.347	-1.15 to 0.42	0.190
Working memory **	0.01 (0.65)	0.81 (0.98)	-2.427	0.024	-1.49 to -0.12	0.437
Learning and memory	-1.02 (1.14)	-1.00 (1.39)	-0.037	0.971	-1.09 to 1.05	0.009
Memory retrieval	-0.92 (1.11)	-0.84 (1.33)	-0.167	0.869	-1.12 to 0.95	0.036
Executive functions	-1.22 (1.60)	-1.54 (1.38)	0.516	0.611	-0.98 to 1.62	0.129
Processing speed	-0.88 (1.35)	-0.28 (0.99)	-1.104	0.283	-1.74 to 0.54	0.274
Global neurocognition	-0.73 (0.88)	-0.47 (0.87)	-0.737	0.469	-1.02 to 0.49	0.137
***Emotional outcomes**, raw score (mean, SD)*						
HADS Anxiety	3.77 (4.53)	4.40 (2.91)	-0.683	0.503	-0.40 to 0.21	0.161
HADS Depression	2.69 (2.96)	6.00 (4.97)	-0.884	0.389	-0.43 to 0.18	0.820
Davidson Trauma Scale	7.38 (9.68)	6.50 (8.75)	-0.095	0.926	-0.45 to 0.41	0.143

ICU Intensive care unit, *TAU* Treatment as usual, *ENRIC* Early neurocognitive stimulation, *HADS* Hospital anxiety and depression scale. ** $p < 0.01$.
